# High-dose tigecycline use in severe infections

**DOI:** 10.1186/cc12018

**Published:** 2013-03-19

**Authors:** G De Pascale, L Montini, T Spanu, V Bernini, A Occhionero, DL Grieco, M Biancone, P De Santis, ES Tanzarella, SL Cutuli, MA Pennisi, MA Antonelli

**Affiliations:** 1 Sacro Cuore Catholic University, Rome, Italy; 2 Institute of Microbiology, Sacro Cuore Catholic University, Rome, Italy

## Introduction

The aim of this study is to describe the clinical and epidemiological profile of ICU patients receiving tigecycline (TGC) and to evaluate the potential benefits of TGC higher doses.

## Methods

All patients admitted to our ICU between 1 June 2009 and 31 May 2012 who received TGC were evaluated. Cases were excluded when infections were not microbiologically confirmed.

## Results

Over the study period, 100 patients fulfilled the inclusion criteria: 54 in the SD group (50 mg every 12 hours) and 46 in the HD group (100 mg every 12 hours). The SD group and the HD group were not significantly different in terms of age, severity of disease, duration of TGC therapy, rate of concomitant other active antibiotic use and of inadequate empirical antimicrobial therapy (IIAT) (*P *= NS). MDR *A.baumannii *and *K. pneumoniae *were the main pathogens isolated. The percentage of germs other than *A. baumannii *and *K. pneumoniae *was higher in the SD TGC group (*P <*0.01). Otherwise infections due to less susceptible germs (TGC MIC value ≥1 μg/ml) were mainly treated with TGC higher doses (*P <*0.01). No significant differences were found in terms of ICU mortality (*P *= 0.8). The rate of abnormal laboratory measures during TGC treatment was similar between the two groups (*P *= NS). No patients required TGC discontinuation or dose reduction because of suspected adverse events. In the VAP subpopulation (63 patients: 30 received SD and 33 HD), the clinical cure rate and microbiological eradication percentage were higher when TGC was used at higher doses (57.6% vs. 33.3%; *P *= 0.08 and 57.1% vs. 30.4%; *P *= 0.1). Table 1 shows multivariate analysis of clinical cure predictors in the VAP subgroup.

**Table 1:** Predictors of clinical cure in patients with VAP

	OR (95% CI)	*P *value
HD TGC	5.93 (1.38 to 25.6)	0.02
IIAT	0.23 (0.06 to 0.85)	0.03
SOFA score	0.62 (0.45 to 0.86)	<0.01

## Conclusion

In critically ill patients, HD TGC use seems to be safe and, combined with other active antibiotics, may increase the rate of MDR germ VAP clinical success. IIAT and the severity degree of patients' clinical condition still remain major determinants of VAP treatment failure.
